# 2-(1*H*-Benzimidazol-1-yl)-1-(2-fur­yl)ethanone *O*-ethyl­oxime

**DOI:** 10.1107/S1600536809022892

**Published:** 2009-06-20

**Authors:** Özden Özel Güven, Taner Erdoğan, M. Nawaz Tahir, Tuncer Hökelek

**Affiliations:** a Zonguldak Karaelmas University, Department of Chemistry, 67100 Zonguldak, Turkey; b Sargodha University, Department of Physics, Sargodha, Pakistan; c Hacettepe University, Department of Physics, 06800 Beytepe, Ankara, Turkey

## Abstract

In the mol­ecule of the title compound, C_15_H_15_N_3_O_2_, the planar benzimidazole ring system [maximum deviation = 0.023 (2) Å] is oriented at a dihedral angle of 74.21 (5)° with respect to the furan ring. In the crystal structure, inter­molecular C—H⋯N inter­actions link the mol­ecules into centrosymmetric *R*_2_^2^(18) dimers. In addition, the structure is stabilized by π–π contacts between parallel imidazole rings [centroid–centroid distance = 3.726 (1) Å] and a weak C—H⋯π inter­action.

## Related literature

For general background to oximes and oxime ethers and their biological activity, see: Baji *et al.* (1995 ▶); Bhandari *et al.* (2009 ▶); Emami *et al.* (2002 ▶, 2004 ▶); Milanese *et al.* (2007 ▶); Polak (1982 ▶); Porretta *et al.* (1993 ▶); Ramalingan *et al.* (2006 ▶); Rossello *et al.* (2002 ▶). For related structures, see: Özel Güven *et al.* (2007*a* ▶,*b* ▶, 2009*a* ▶,*b* ▶). For ring-motifs, see: Bernstein *et al.* (1995 ▶). 
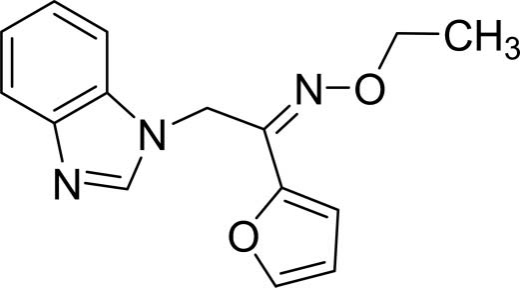


## Experimental

### 

#### Crystal data

C_15_H_15_N_3_O_2_
*M* *_r_* = 269.30
Monoclinic, 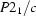

*a* = 8.4448 (5) Å
*b* = 17.6345 (11) Å
*c* = 10.3147 (6) Å
β = 110.755 (2)°
*V* = 1436.38 (15) Å^3^
*Z* = 4
Mo *K*α radiation
μ = 0.09 mm^−1^
*T* = 296 K
0.40 × 0.25 × 0.20 mm

#### Data collection

Bruker Kappa APEXII CCD diffractometer
Absorption correction: multi-scan (*SADABS*; Bruker, 2005 ▶) *T*_min_ = 0.967, *T*_max_ = 0.979
16676 measured reflections
3742 independent reflections
2291 reflections with *I* > 2σ(*I*)
*R*_int_ = 0.027

#### Refinement

*R*[*F* ^2^ > 2σ(*F* ^2^)] = 0.047
*wR*(*F* ^2^) = 0.140
*S* = 1.03
3742 reflections
182 parameters
H-atom parameters constrained
Δρ_max_ = 0.19 e Å^−3^
Δρ_min_ = −0.18 e Å^−3^

### 

Data collection: *APEX2* (Bruker, 2007 ▶); cell refinement: *SAINT* (Bruker, 2007 ▶); data reduction: *SAINT*; program(s) used to solve structure: *SHELXS97* (Sheldrick, 2008 ▶); program(s) used to refine structure: *SHELXL97* (Sheldrick, 2008 ▶); molecular graphics: *ORTEP-3 for Windows* (Farrugia, 1997 ▶); software used to prepare material for publication: *WinGX* (Farrugia, 1999 ▶) and *PLATON* (Spek, 2009 ▶).

## Supplementary Material

Crystal structure: contains datablocks I, global. DOI: 10.1107/S1600536809022892/xu2539sup1.cif

Structure factors: contains datablocks I. DOI: 10.1107/S1600536809022892/xu2539Isup2.hkl

Additional supplementary materials:  crystallographic information; 3D view; checkCIF report

## Figures and Tables

**Table 1:** Hydrogen-bond geometry (Å, °)

*D*—H⋯*A*	*D*—H	H⋯*A*	*D*⋯*A*	*D*—H⋯*A*
C13—H13⋯N2^i^	0.93	2.54	3.328 (2)	143
C14—H14*A*⋯*Cg*2^ii^	0.97	2.88	3.768 (2)	153

Symmetry codes: (i) 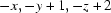; (ii) 
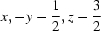
.*Cg*2 is the centroid of the C2–C7 ring.

**Fig. 1.**
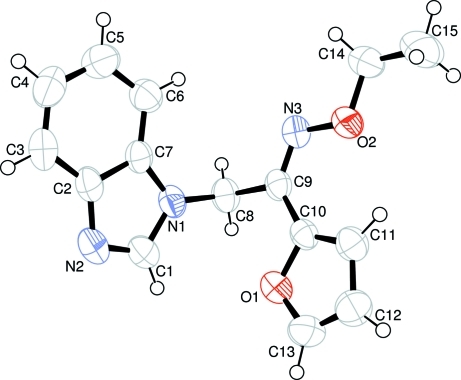
The molecular structure of the title molecule with the atom-numbering scheme. Displacement ellipsoids are drawn at the 50% probability level.

**Fig. 2.**
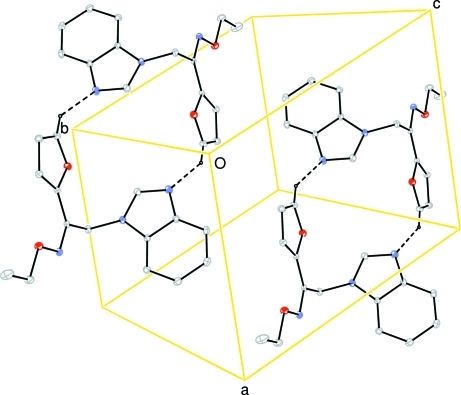
A partial packing diagram of the title compound. Hydrogen bonds are shown as dashed lines. H atoms not involved in hydrogen bonding have been omitted for clarity.

## supplementary crystallographic information

### Comment

Oximes and oxime ethers show very important antifungal and antibacterial activities. Oxiconazole is a well established drug for treatment of many mycotic infections, having an oxime group (Polak, 1982). Several compounds containing an oxime or an oxime ether function have been reported to exhibit antimicrobial activity (Porretta *et al.*, 1993; Baji *et al.*, 1995; Rossello *et al.*, 2002; Emami *et al.*, 2002; Emami *et al.*, 2004; Ramalingan *et al.*, 2006; Milanese *et al.*, 2007; Bhandari *et al.*, 2009). In our earlier studies, we reported X-ray structures of benzimidazole substituted oxiconazole derivatives (Özel Güven *et al.*, 2007*a*; Özel Güven *et al.*, 2009*a*; Özel Güven *et al.*, 2009*b*). Now, we report herein the crystal structure of the title alkyl oxime ether.

In the molecule of the title compound (Fig. 1), the bond lengths and angles are generally within normal ranges. The planar benzimidazole ring system [with a maximum deviation of 0.023 (2) Å for atom C5] is oriented with respect to the furan ring at a dihedral angle of 74.21 (5)°. Atoms C8, C9 and N3 are -0.066 (2), 0.001 (1) and 0.055 (1) Å away from the furan ring plane, respectively, while atom C8 is at a distance of 0.006 (2) Å to the benzimidazole ring plane. So, they are coplanar with the adjacent rings. The N1—C1—N2 [114.46 (16)°], N2—C2—C7 [110.10 (15)°], C2—C7—C6 [122.57 (15)°], C3—C4—C5 [121.26 (18)°] and C4—C5—C6 [121.86 (18)°] bond angles are enlarged, while C5—C6—C7 [116.19 (17)°] and C2—C3—C4 [118.29 (17)°] bond angles are narrowed.

In the crystal structure, intermolecular C—H···N interactions (Table 1) link the molecules into centrosymmetric dimers through *R*_2_^2^(18) ring motifs (Bernstein *et al.*, 1995) (Fig. 2), in which they may be effective in the stabilization of the structure. The π–π contact between the imidazole rings, *Cg*1—*Cg*1^i^, [symmetry code: (i) 1 - *x*, -*y*, 1 - *z*, where *Cg*1 is centroid of the ring (N1/N2/C1/C2/C7)] may further stabilize the structure, with centroid-centroid distance of 3.726 (1) Å. A weak C—H···π interaction (Table 1) is also found.

### Experimental

The title compound was synthesized by the reaction of 2-(1*H*-benzimidazol-1-yl)-1-(furan-2-yl)ethanone oxime obtained from 2-(1*H*-benzimidazol-1-yl)-1-(furan-2-yl)ethanone (Özel Güven *et al.*, 2007*b*) with ethyl iodide and NaH. To a solution of 2-(1*H*-benzimidazol-1-yl)-1-(furan-2-yl)ethanone oxime (400 mg, 1.658 mmol) in DMF (5 ml) was added NaH (66 mg, 1.658 mmol) in small fractions. Then, ethyl iodide (259 mg, 1.658 mmol) was added dropwise. The mixture was stirred at room temperature for 3 h and the excess of hydride was decomposed with a small amount of methyl alcohol. After evaporation to dryness under reduced pressure, the crude residue was suspended with water and extracted with methylene chloride. The organic layer was dried over anhydrous sodium sulfate and then evaporated to dryness. The crude residue was purified by chromatography on a silica-gel column using chloroform and recrystallized from hexane-ethyl acetate (1:3) mixture to obtain yellow crystals (yield; 270 mg, 61%).

### Refinement

H atoms were positioned geometrically with C—H = 0.93, 0.97 and 0.96 Å, for aromatic, methylene and methyl H atoms and constrained to ride on their parent atoms, with *U*_iso_(H) = *xU*_eq_(C), where *x* = 1.5 for methyl H and *x* = 1.2 for all other H atoms.

### Crystal data

**Table:**

C_15_H_15_N_3_O_2_	*F*(000) = 568
*M**_r_* = 269.30	*D*_x_ = 1.245 Mg m^−^^3^
Monoclinic, *P*2_1_/*c*	Mo *K*α radiation, λ = 0.71073 Å
Hall symbol: -P 2ybc	Cell parameters from 1232 reflections
*a* = 8.4448 (5) Å	θ = 2.3–28.8°
*b* = 17.6345 (11) Å	µ = 0.09 mm^−^^1^
*c* = 10.3147 (6) Å	*T* = 296 K
β = 110.755 (2)°	Block, yellow
*V* = 1436.38 (15) Å^3^	0.40 × 0.25 × 0.20 mm
*Z* = 4	

### Data collection

**Table:**

Bruker Kappa APEXII CCD diffractometer	3742 independent reflections
Radiation source: fine-focus sealed tube	2291 reflections with *I* > 2σ(*I*)
graphite	*R*_int_ = 0.027
ω scans	θ_max_ = 28.8°, θ_min_ = 2.3°
Absorption correction: multi-scan (*SADABS*; Bruker, 2005)	*h* = −11→7
*T*_min_ = 0.967, *T*_max_ = 0.979	*k* = −23→19
16676 measured reflections	*l* = −12→13

### Refinement

**Table:**

Refinement on *F*^2^	Primary atom site location: structure-invariant direct methods
Least-squares matrix: full	Secondary atom site location: difference Fourier map
*R*[*F*^2^ > 2σ(*F*^2^)] = 0.047	Hydrogen site location: inferred from neighbouring sites
*wR*(*F*^2^) = 0.140	H-atom parameters constrained
*S* = 1.02	*w* = 1/[σ^2^(*F*_o_^2^) + (0.0597*P*)^2^ + 0.249*P*] where *P* = (*F*_o_^2^ + 2*F*_c_^2^)/3
3742 reflections	(Δ/σ)_max_ < 0.001
182 parameters	Δρ_max_ = 0.19 e Å^−^^3^
0 restraints	Δρ_min_ = −0.18 e Å^−^^3^

### Special details

**Table:**

Geometry. All e.s.d.'s (except the e.s.d. in the dihedral angle between two l.s. planes) are estimated using the full covariance matrix. The cell e.s.d.'s are taken into account individually in the estimation of e.s.d.'s in distances, angles and torsion angles; correlations between e.s.d.'s in cell parameters are only used when they are defined by crystal symmetry. An approximate (isotropic) treatment of cell e.s.d.'s is used for estimating e.s.d.'s involving l.s. planes.
Refinement. Refinement of *F*^2^ against ALL reflections. The weighted *R*-factor *wR* and goodness of fit *S* are based on *F*^2^, conventional *R*-factors *R* are based on *F*, with *F* set to zero for negative *F*^2^. The threshold expression of *F*^2^ > σ(*F*^2^) is used only for calculating *R*-factors(gt) *etc*. and is not relevant to the choice of reflections for refinement. *R*-factors based on *F*^2^ are statistically about twice as large as those based on *F*, and *R*- factors based on ALL data will be even larger.

### Fractional atomic coordinates and isotropic or equivalent isotropic displacement parameters (Å^2^)

**Table:**

	*x*	*y*	*z*	*U*_iso_*/*U*_eq_	
O1	−0.07624 (14)	0.47405 (6)	0.69126 (11)	0.0587 (3)	
O2	0.12331 (14)	0.34870 (6)	0.45867 (12)	0.0620 (3)	
N1	0.30309 (17)	0.45287 (7)	0.87568 (13)	0.0525 (3)	
N2	0.2926 (2)	0.43475 (9)	1.08660 (15)	0.0735 (5)	
N3	0.23041 (17)	0.39663 (8)	0.55862 (13)	0.0544 (3)	
C1	0.2331 (2)	0.47207 (11)	0.97034 (18)	0.0672 (5)	
H1	0.1494	0.5089	0.9538	0.081*	
C2	0.4123 (2)	0.38660 (10)	1.06784 (16)	0.0561 (4)	
C3	0.5178 (2)	0.33399 (11)	1.15764 (18)	0.0691 (5)	
H3	0.5126	0.3257	1.2451	0.083*	
C4	0.6289 (3)	0.29490 (12)	1.1144 (2)	0.0752 (5)	
H4	0.7002	0.2594	1.1732	0.090*	
C5	0.6378 (2)	0.30710 (12)	0.9839 (2)	0.0756 (5)	
H5	0.7168	0.2802	0.9583	0.091*	
C6	0.5329 (2)	0.35802 (10)	0.89142 (19)	0.0629 (4)	
H6	0.5373	0.3655	0.8035	0.076*	
C7	0.42077 (19)	0.39732 (8)	0.93684 (15)	0.0489 (4)	
C8	0.2629 (2)	0.48404 (9)	0.73688 (16)	0.0545 (4)	
H8A	0.3672	0.4931	0.7200	0.065*	
H8B	0.2060	0.5324	0.7313	0.065*	
C9	0.15179 (19)	0.43200 (8)	0.62662 (14)	0.0461 (3)	
C10	−0.02592 (19)	0.42668 (8)	0.60758 (15)	0.0460 (3)	
C11	−0.1609 (2)	0.38634 (9)	0.52731 (17)	0.0564 (4)	
H11	−0.1616	0.3501	0.4616	0.068*	
C12	−0.3007 (2)	0.40962 (11)	0.5621 (2)	0.0674 (5)	
H12	−0.4112	0.3918	0.5237	0.081*	
C13	−0.2438 (2)	0.46193 (11)	0.6602 (2)	0.0679 (5)	
H13	−0.3102	0.4869	0.7020	0.081*	
C14	0.2185 (2)	0.31439 (13)	0.3830 (2)	0.0813 (6)	
H14A	0.3135	0.2863	0.4460	0.098*	
H14B	0.2625	0.3534	0.3386	0.098*	
C15	0.1095 (3)	0.26391 (19)	0.2798 (3)	0.1340 (12)	
H15A	0.1742	0.2382	0.2331	0.201*	
H15B	0.0206	0.2926	0.2136	0.201*	
H15C	0.0610	0.2273	0.3238	0.201*	

### Atomic displacement parameters (Å^2^)

**Table:**

	*U*^11^	*U*^22^	*U*^33^	*U*^12^	*U*^13^	*U*^23^
O1	0.0639 (7)	0.0630 (7)	0.0511 (6)	0.0017 (5)	0.0226 (5)	−0.0089 (5)
O2	0.0581 (7)	0.0677 (7)	0.0577 (7)	0.0012 (6)	0.0173 (5)	−0.0193 (6)
N1	0.0589 (8)	0.0543 (7)	0.0408 (7)	−0.0030 (6)	0.0136 (6)	−0.0069 (6)
N2	0.0865 (11)	0.0894 (11)	0.0453 (8)	0.0104 (9)	0.0241 (8)	−0.0082 (8)
N3	0.0554 (7)	0.0591 (8)	0.0453 (7)	−0.0013 (6)	0.0136 (6)	−0.0042 (6)
C1	0.0761 (12)	0.0722 (11)	0.0518 (10)	0.0112 (9)	0.0210 (9)	−0.0116 (9)
C2	0.0597 (9)	0.0634 (10)	0.0400 (8)	−0.0069 (8)	0.0112 (7)	−0.0092 (7)
C3	0.0748 (12)	0.0795 (12)	0.0429 (9)	−0.0053 (10)	0.0083 (8)	0.0017 (8)
C4	0.0676 (11)	0.0768 (12)	0.0647 (12)	0.0044 (10)	0.0029 (10)	0.0055 (10)
C5	0.0636 (11)	0.0796 (13)	0.0809 (14)	0.0112 (10)	0.0222 (10)	−0.0037 (11)
C6	0.0611 (10)	0.0722 (11)	0.0578 (10)	−0.0017 (9)	0.0239 (8)	−0.0043 (9)
C7	0.0477 (8)	0.0516 (8)	0.0420 (8)	−0.0091 (7)	0.0094 (6)	−0.0069 (6)
C8	0.0619 (10)	0.0515 (9)	0.0470 (9)	−0.0101 (7)	0.0153 (7)	−0.0003 (7)
C9	0.0551 (9)	0.0438 (7)	0.0374 (8)	−0.0002 (6)	0.0141 (6)	0.0044 (6)
C10	0.0577 (9)	0.0420 (7)	0.0389 (8)	0.0011 (6)	0.0180 (6)	0.0014 (6)
C11	0.0615 (10)	0.0527 (9)	0.0552 (9)	−0.0061 (7)	0.0210 (8)	−0.0052 (7)
C12	0.0580 (10)	0.0751 (11)	0.0709 (12)	−0.0095 (9)	0.0252 (9)	−0.0033 (10)
C13	0.0632 (11)	0.0810 (12)	0.0684 (12)	0.0060 (9)	0.0342 (9)	0.0004 (10)
C14	0.0680 (12)	0.0984 (15)	0.0776 (13)	0.0098 (11)	0.0257 (10)	−0.0302 (12)
C15	0.0854 (16)	0.165 (3)	0.138 (2)	0.0083 (17)	0.0234 (16)	−0.097 (2)

### Geometric parameters (Å, °)

**Table:**

O1—C10	1.3723 (17)	C7—C6	1.382 (2)
O1—C13	1.352 (2)	C8—H8A	0.9700
O2—N3	1.3909 (16)	C8—H8B	0.9700
O2—C14	1.438 (2)	C9—N3	1.285 (2)
N1—C1	1.351 (2)	C9—C8	1.503 (2)
N1—C7	1.379 (2)	C9—C10	1.446 (2)
N1—C8	1.457 (2)	C10—C11	1.350 (2)
N2—C1	1.303 (2)	C11—C12	1.411 (2)
C1—H1	0.9300	C11—H11	0.9300
C2—N2	1.385 (2)	C12—H12	0.9300
C2—C3	1.388 (2)	C13—C12	1.328 (3)
C3—C4	1.361 (3)	C13—H13	0.9300
C3—H3	0.9300	C14—C15	1.442 (3)
C4—H4	0.9300	C14—H14A	0.9700
C5—C4	1.391 (3)	C14—H14B	0.9700
C5—H5	0.9300	C15—H15A	0.9600
C6—C5	1.379 (3)	C15—H15B	0.9600
C6—H6	0.9300	C15—H15C	0.9600
C7—C2	1.391 (2)		
			
C13—O1—C10	106.63 (13)	C9—C8—H8A	109.2
N3—O2—C14	108.44 (12)	C9—C8—H8B	109.2
C1—N1—C7	106.03 (13)	H8A—C8—H8B	107.9
C1—N1—C8	127.31 (15)	N3—C9—C8	113.87 (14)
C7—N1—C8	126.65 (13)	N3—C9—C10	127.33 (14)
C1—N2—C2	104.09 (14)	C10—C9—C8	118.81 (13)
C9—N3—O2	111.93 (13)	O1—C10—C9	114.39 (13)
N1—C1—H1	122.8	C11—C10—O1	108.92 (13)
N2—C1—N1	114.46 (16)	C11—C10—C9	136.69 (14)
N2—C1—H1	122.8	C10—C11—C12	106.94 (15)
N2—C2—C3	130.09 (16)	C10—C11—H11	126.5
N2—C2—C7	110.10 (15)	C12—C11—H11	126.5
C3—C2—C7	119.80 (16)	C11—C12—H12	126.7
C2—C3—H3	120.9	C13—C12—C11	106.65 (16)
C4—C3—C2	118.29 (17)	C13—C12—H12	126.7
C4—C3—H3	120.9	O1—C13—H13	124.6
C3—C4—C5	121.26 (18)	C12—C13—O1	110.85 (16)
C3—C4—H4	119.4	C12—C13—H13	124.6
C5—C4—H4	119.4	O2—C14—C15	109.09 (17)
C4—C5—H5	119.1	O2—C14—H14A	109.9
C6—C5—C4	121.86 (18)	O2—C14—H14B	109.9
C6—C5—H5	119.1	C15—C14—H14A	109.9
C5—C6—C7	116.19 (17)	C15—C14—H14B	109.9
C5—C6—H6	121.9	H14A—C14—H14B	108.3
C7—C6—H6	121.9	C14—C15—H15A	109.5
N1—C7—C6	132.10 (15)	C14—C15—H15B	109.5
N1—C7—C2	105.31 (14)	C14—C15—H15C	109.5
C6—C7—C2	122.57 (15)	H15A—C15—H15B	109.5
N1—C8—C9	112.26 (12)	H15A—C15—H15C	109.5
N1—C8—H8A	109.2	H15B—C15—H15C	109.5
N1—C8—H8B	109.2		
			
C13—O1—C10—C9	179.95 (13)	C7—C6—C5—C4	1.3 (3)
C13—O1—C10—C11	−0.15 (17)	N1—C7—C2—N2	−0.25 (18)
C10—O1—C13—C12	0.1 (2)	N1—C7—C2—C3	−179.41 (14)
C14—O2—N3—C9	177.14 (15)	C6—C7—C2—N2	178.33 (15)
N3—O2—C14—C15	179.4 (2)	C6—C7—C2—C3	−0.8 (2)
C7—N1—C1—N2	−0.1 (2)	N1—C7—C6—C5	177.93 (16)
C8—N1—C1—N2	179.70 (15)	C2—C7—C6—C5	−0.2 (2)
C1—N1—C7—C2	0.23 (17)	C8—C9—N3—O2	179.48 (11)
C1—N1—C7—C6	−178.15 (17)	C10—C9—N3—O2	−0.6 (2)
C8—N1—C7—C2	−179.61 (14)	N3—C9—C8—N1	−104.67 (16)
C8—N1—C7—C6	2.0 (3)	C10—C9—C8—N1	75.41 (17)
C1—N1—C8—C9	−102.12 (19)	N3—C9—C10—O1	−177.03 (14)
C7—N1—C8—C9	77.69 (19)	N3—C9—C10—C11	3.1 (3)
C2—N2—C1—N1	0.0 (2)	C8—C9—C10—O1	2.88 (18)
C3—C2—N2—C1	179.21 (18)	C8—C9—C10—C11	−176.98 (17)
C7—C2—N2—C1	0.16 (19)	O1—C10—C11—C12	0.16 (18)
N2—C2—C3—C4	−178.13 (18)	C9—C10—C11—C12	−179.97 (17)
C7—C2—C3—C4	0.8 (3)	C10—C11—C12—C13	−0.1 (2)
C2—C3—C4—C5	0.2 (3)	O1—C13—C12—C11	0.0 (2)
C6—C5—C4—C3	−1.3 (3)		

### Hydrogen-bond geometry (Å, °)

**Table:**

*D*—H···*A*	*D*—H	H···*A*	*D*···*A*	*D*—H···*A*
C13—H13···N2^i^	0.93	2.54	3.328 (2)	143
C14—H14A···Cg2^ii^	0.97	2.88	3.768 (2)	153

Symmetry codes: (i) −*x*, −*y*+1, −*z*+2; (ii) *x*, −*y*−1/2, *z*−3/2.
